# Generative artificial intelligence to produce high-fidelity blastocyst-stage embryo images

**DOI:** 10.1093/humrep/deae064

**Published:** 2024-04-10

**Authors:** Ping Cao, Josien Derhaag, Edith Coonen, Han Brunner, Ganesh Acharya, Andres Salumets, Masoud Zamani Esteki

**Affiliations:** Department of Clinical Genetics, Maastricht University Medical Center+ (MUMC+), Maastricht, The Netherlands; Department of Genetics and Cell Biology, GROW Research Institute for Oncology and Reproduction, Faculty of Health, Medicine and Life Sciences (FHML), Maastricht University, Maastricht, The Netherlands; Department of Reproductive Medicine, Maastricht University Medical Center+ (MUMC+), Maastricht, The Netherlands; Department of Clinical Genetics, Maastricht University Medical Center+ (MUMC+), Maastricht, The Netherlands; Department of Reproductive Medicine, Maastricht University Medical Center+ (MUMC+), Maastricht, The Netherlands; Department of Clinical Genetics, Maastricht University Medical Center+ (MUMC+), Maastricht, The Netherlands; Department of Genetics and Cell Biology, GROW Research Institute for Oncology and Reproduction, Faculty of Health, Medicine and Life Sciences (FHML), Maastricht University, Maastricht, The Netherlands; Department of Human Genetics, Radboud University Medical Center, Nijmegen, The Netherlands; Division of Obstetrics and Gynecology, Department of Clinical Science, Intervention and Technology (CLINTEC), Karolinska Institutet, and Karolinska University Hospital, Stockholm, Sweden; Women’s Health and Perinatology Research Group, Department of Clinical Medicine, UiT—The Arctic University of Norway, Tromsø, Norway; Division of Obstetrics and Gynecology, Department of Clinical Science, Intervention and Technology (CLINTEC), Karolinska Institutet, and Karolinska University Hospital, Stockholm, Sweden; Competence Centre on Health Technologies, Tartu, Estonia; Department of Obstetrics and Gynecology, Institute of Clinical Medicine, University of Tartu, Tartu, Estonia; Department of Clinical Genetics, Maastricht University Medical Center+ (MUMC+), Maastricht, The Netherlands; Department of Genetics and Cell Biology, GROW Research Institute for Oncology and Reproduction, Faculty of Health, Medicine and Life Sciences (FHML), Maastricht University, Maastricht, The Netherlands; Division of Obstetrics and Gynecology, Department of Clinical Science, Intervention and Technology (CLINTEC), Karolinska Institutet, and Karolinska University Hospital, Stockholm, Sweden

**Keywords:** artificial intelligence, generative adversarial networks, IVF, time-lapse microscopy, embryo images, assisted reproductive technology, reproductive medicine, embryogenesis, image processing

## Abstract

**STUDY QUESTION:**

Can generative artificial intelligence (AI) models produce high-fidelity images of human blastocysts?

**SUMMARY ANSWER:**

Generative AI models exhibit the capability to generate high-fidelity human blastocyst images, thereby providing substantial training datasets crucial for the development of robust AI models.

**WHAT IS KNOWN ALREADY:**

The integration of AI into IVF procedures holds the potential to enhance objectivity and automate embryo selection for transfer. However, the effectiveness of AI is limited by data scarcity and ethical concerns related to patient data privacy. Generative adversarial networks (GAN) have emerged as a promising approach to alleviate data limitations by generating synthetic data that closely approximate real images.

**STUDY DESIGN, SIZE, DURATION:**

Blastocyst images were included as training data from a public dataset of time-lapse microscopy (TLM) videos (n = 136). A style-based GAN was fine-tuned as the generative model.

**PARTICIPANTS/MATERIALS, SETTING, METHODS:**

We curated a total of 972 blastocyst images as training data, where frames were captured within the time window of 110–120 h post-insemination at 1-h intervals from TLM videos. We configured the style-based GAN model with data augmentation (AUG) and pretrained weights (Pretrained-T: with translation equivariance; Pretrained-R: with translation and rotation equivariance) to compare their optimization on image synthesis. We then applied quantitative metrics including Fréchet Inception Distance (FID) and Kernel Inception Distance (KID) to assess the quality and fidelity of the generated images. Subsequently, we evaluated qualitative performance by measuring the intelligence behavior of the model through the visual Turing test. To this end, 60 individuals with diverse backgrounds and expertise in clinical embryology and IVF evaluated the quality of synthetic embryo images.

**MAIN RESULTS AND THE ROLE OF CHANCE:**

During the training process, we observed consistent improvement of image quality that was measured by FID and KID scores. Pretrained and AUG + Pretrained initiated with remarkably lower FID and KID values compared to both Baseline and AUG + Baseline models. Following 5000 training iterations, the AUG + Pretrained-R model showed the highest performance of the evaluated five configurations with FID and KID scores of 15.2 and 0.004, respectively. Subsequently, we carried out the visual Turing test, such that IVF embryologists, IVF laboratory technicians, and non-experts evaluated the synthetic blastocyst-stage embryo images and obtained similar performance in specificity with marginal differences in accuracy and sensitivity.

**LIMITATIONS, REASONS FOR CAUTION:**

In this study, we primarily focused the training data on blastocyst images as IVF embryos are primarily assessed in blastocyst stage. However, generation of an array of images in different preimplantation stages offers further insights into the development of preimplantation embryos and IVF success. In addition, we resized training images to a resolution of 256 × 256 pixels to moderate the computational costs of training the style-based GAN models. Further research is needed to involve a more extensive and diverse dataset from the formation of the zygote to the blastocyst stage, e.g. video generation, and the use of improved image resolution to facilitate the development of comprehensive AI algorithms and to produce higher-quality images.

**WIDER IMPLICATIONS OF THE FINDINGS:**

Generative AI models hold promising potential in generating high-fidelity human blastocyst images, which allows the development of robust AI models as it can provide sufficient training datasets while safeguarding patient data privacy. Additionally, this may help to produce sufficient embryo imaging training data with different (rare) abnormal features, such as embryonic arrest, tripolar cell division to avoid class imbalances and reach to even datasets. Thus, generative models may offer a compelling opportunity to transform embryo selection procedures and substantially enhance IVF outcomes.

**STUDY FUNDING/COMPETING INTEREST(S):**

This study was supported by a Horizon 2020 innovation grant (ERIN, grant no. EU952516) and a Horizon Europe grant (NESTOR, grant no. 101120075) of the European Commission to A.S. and M.Z.E., the Estonian Research Council (grant no. PRG1076) to A.S., and the EVA (Erfelijkheid Voortplanting & Aanleg) specialty program (grant no. KP111513) of Maastricht University Medical Centre (MUMC+) to M.Z.E.

**TRIAL REGISTRATION NUMBER:**

Not applicable.

## Introduction

Traditionally IVF involves selecting embryos based on a few microscopic observations or static images captured at specific timepoints during post-insemination development (De los Santos *et al.*, 2016). However, these approaches capture only limited information about the dynamic changes that occur during early *in vitro* embryogenesis. The implementation of time-lapse microscopy (TLM) in human embryology (IVF laboratories) provides a continuous temporal overview of preimplantation development, including the pace and evenness of cell divisions, timepoints of developmental events such as morula or blastocyst formation that can be indicative of normal or abnormal preimplantation development and pregnancy outcome (Wong *et al.*, 2010). Although embryo morphology scoring has been the standard practice for IVF embryo selection, it is important to note that visual inspections by clinical embryologists introduce subjectivity, diminishing reproducibility, and predictive accuracy for successful pregnancy (Storr *et al.*, 2017), which is primarily due to limited visibility of embryonic features in 2D images and that microscopic image analyses only capture a specific timepoint of the development. For instance, it has been shown that counting pronuclei would not necessarily differentiate an abnormal embryo from a normal embryo both in terms of morphology and genome integrity (Destouni *et al.*, 2018). Therefore, artificial intelligence (AI)-based automatic annotations, characterized by high accuracy and reliability, which capture and evaluate the continuous development of IVF embryos can enhance embryo selection strategies and prediction of IVF success (Jiang and Bormann, 2023).

The incorporation of AI into medical imaging has seen remarkable progress in the areas of automation of medical processes and the development of precision medicine (Zhou *et al.*, 2021; Chen *et al.*, 2022). Nonetheless, AI-driven methods heavily rely on abundant labeled data, a requirement often met with difficulties in many medical imaging datasets, including severe class imbalance where the distribution of labeled data is uneven (Miotto *et al.*, 2017). To address these challenges, generative adversarial networks (GAN) have been developed (Goodfellow *et al.*, 2014), such that synthetic images that closely resemble the distribution of real data could be generated (Kazeminia *et al.*, 2020). GAN has been proven to have a wide range of capabilities in medical imaging, including image synthesis, segmentation, reconstruction, detection, and classification tasks (Kazeminia *et al.*, 2020), as well as domain shifting between the source and target datasets from different centers with different imaging systems (Kanakasabapathy *et al.*, 2021). In general, GAN has broader applications in image synthesis, which can be categorized into two modes: unconditional and conditional. Unconditional training mirrors the original GAN model, functioning in an unsupervised manner, and generating data primarily from a noise vector, with limited influence on the output. Deep convolutional GAN (DCGAN) (Radford *et al.*, 2016), leveraging convolutional networks, has played a substantial role in serving as both unconditional and conditional training models. For example, the combination of real and generated images to train DCGAN led to improved pathology classification of chest X-rays when compared to networks trained solely on real images (Salehinejad *et al.*, 2019). Additionally, the BrainGAN (Alrashedy *et al.*, 2022) framework proposed a fusion of original GAN and DCGAN for generating brain magnetic resonance (MR) images, along with an automatic way to classify the generated images. Conditional GAN (cGAN) (Mirza and Osindero, 2014) integrates valuable prior information as the condition into the training process, allowing for greater control over the generation procedure. For instance, generating cardiac MR images with heart tissue labels for segmentation analysis (Al Khalil *et al.*, 2023). Another approach, CycleGAN (Zhu *et al.*, 2017), uses image-to-image translation with unpaired data, streamlining the training process, particularly in scenarios with limited data and desired classes. Notably, the application of CycleGAN for augmentation (AUG) with lung computed tomography (CT) slices has demonstrated significant enhancements in the performance of deep learning models for automatic diagnosis (Ghassemi *et al.*, 2023).

A style-based generator for GAN (StyleGAN) (Karras *et al.*, 2018) adjusts the ‘style’ of the embedded image features at every convolution layer, representing a state-of-the-art generative model for generating high-resolution images. It enables progressive training with varying input resolutions and noise injection into network, leading to improved image quality and training stability. For instance, using StyleGAN to augment microfluidic chip images demonstrated significantly enhanced accuracy of mobile health diagnostics for infectious diseases (Shokr *et al.*, 2021). Additionally, StyleGAN has been successfully applied in medical imaging by transforming latent style vectors between CT and MR for cross-modality analysis (Fetty *et al.*, 2020). A previous study, HEMIGEN (Dirvanauskas *et al.*, 2019) employed GAN to generate images representing different stages of human embryo development, including one, two-, and four-cell cleavage stage embryos. While this study showed promise in aiding the advancement of novel algorithms designed for processing embryo images, the specific GAN architecture is limited to producing 200 × 200 pixels images. Additionally, no 8-cell cleavage stage embryos or blastocysts were included, as Day 3 and Day 5 embryos are the primarily used timepoints for clinical morphological assessment in IVF.

In this study, we assessed the ability of generative models to synthesize human embryo images. To this end, we assessed a style-based GAN model performance by leveraging pretrained weights and data AUG, to generate a sufficient number of blastocyst-stage embryo images and assess their quality and fidelity compared to real human embryo images.

## Materials and methods

### Training data curation

**Figure 1.**
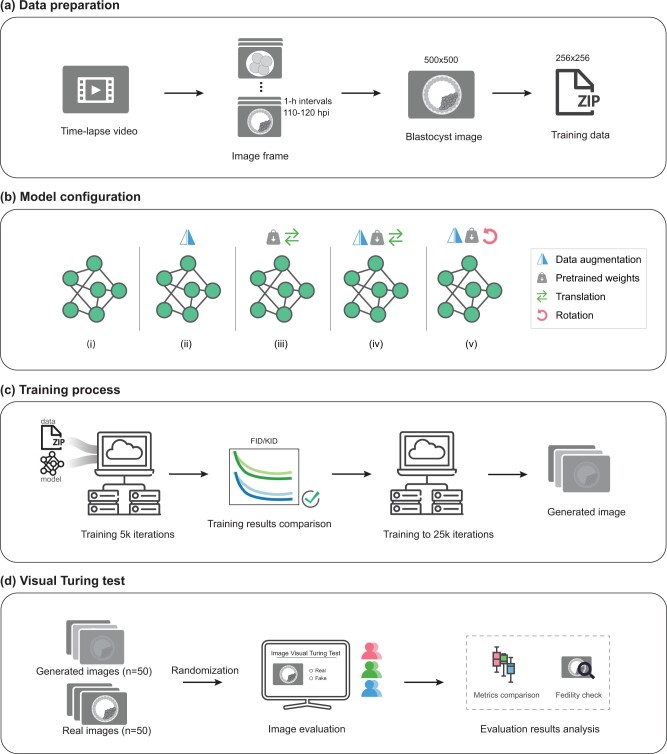
**Schematic overview of the study**. (**a**) Blastocyst images from 110 to 120  hpi were extracted with 1-h intervals from embryo time-lapse videos. Training images were resized before feeding into the generative model. (**b**) Model configurations from left to right: (i) Baseline, the original StyleGAN3 model with randomly initiated training weights. (ii) Pretrained-T, model equipped with pretrained weights with translation equivariance. (iii) AUG + Baseline, data augmented training on Baseline model. (iv) AUG + Pretrained-T, data augmented and pretrained model with translation equivariance. (v) AUG + Pretrained-R, data augmented and pretrained model with translation and rotation equivariance. (**c**) Models were trained at 5000 iterations to compare their FID and KID values. The best model was continued training till 25 000 iterations to generate high-fidelity images. (**d**) Randomly selected real images (n = 50) and fake images (n = 50) were assessed by visual Turing test, i.e. human evaluators, through an online survey. These evaluating metric results were analyzed subsequently.

Given the limited number of publicly available datasets, the training data for this study was obtained from an accessible TLM dataset (Fordham *et al.*, 2022), which was captured using the EmbryoScope^®^ time-lapse system (Vitrolife, Sweden). To acquire blastocyst images, frames captured within the time window of 110–120 hours post-insemination (hpi), with 1-h intervals, were included. This time window was chosen due to its relevance to the developmental stage of embryos, which transitions from the post-morula stage to the blastocyst stage, accounting for temporal differences in blastocyst formation (Alpha Scientists in Reproductive Medicine and ESHRE Special Interest Group of Embryology, 2011; Barnes *et al.*, 2023). Consequently, the training dataset consisted of 972 blastocyst images. The original images were sized at 500 × 500 pixels, and for uniformity, model training, and processing efficiency, all images were resized to 256 × 256 pixels (Fig. 1a).

### Generative adversarial networks

**Figure 2.**
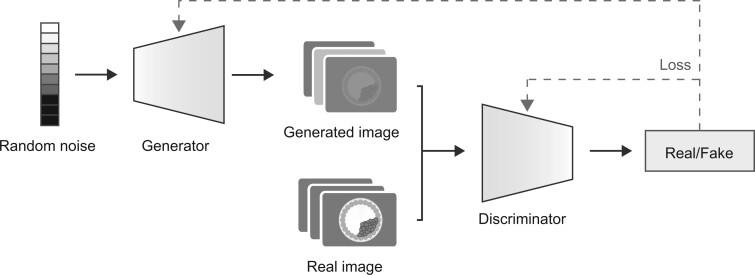
**GAN architecture**. GAN consists of two main modules: a generator and a discriminator. The discriminator aims to distinguish real images from those generated by the generator, while the generator aims to produce synthetic images that effectively deceive the discriminator.

Our GAN model comprises two fundamental components: a discriminator (D) and a generator (G). The objective of the discriminator is to differentiate real images (x) from those produced by the generator (G(z)), while the generator aims to generate synthetic images that effectively deceive the discriminator (Fig. 2). This procedure is directed by the utilization of the cost function:

$$\underset{G}{\min}\;\underset{D}{\;\max\;}\;V(D,G)=E_{x∼p_{\text{data}}(x)}[\log(D(x))]+E_{z∼p_{z}(z)}[1-\log(D(G(z)))]$$

where E represents the expected value, x represents real data, and z represents noise vectors sampled from the probability distribution $p_{z}(z)$.

The primary goal of the discriminator is to maximize the likelihood of precisely categorizing real images D(x) and synthetic images D(G(z))), while the generator aims to reduce the classification error 1-log⁡(D(G(z))). This cost function guides the optimization process of both the discriminator and generator during the adversarial training process. In simpler terms, the discriminator learns to distinguish real from synthetic images, while the generator aims to create synthetic images that are so convincing that they fool the discriminator. This adversarial process leads to the continuous improvement of the generator in producing high-quality synthetic data. The noise vector z injected into the generator represents a source of random variability. It allows the generator to introduce diversity into the synthetic data it produces, enhancing its ability to generate a wide range of realistic images.

In this study, we adapted the StyleGAN3 (Karras *et al.*, 2021) network due to its outstanding performance on generating high-resolution images and diverse samples. Specifically, each module was tailored to a specific purpose: (i) Baseline training (Baseline): Involves training the model with randomly initialized weights, providing a baseline for comparison with other configurations to assess the model’s original performance without prior knowledge. (ii) Pretrained model with translation equivariance (Pretrained-T): Uses pretrained weights from the FFHQ dataset—a diverse human face dataset (Karras *et al.*, 2021) to investigate the benefits of transfer learning. By initializing the model with pretrained weights from another large dataset, it is anticipated that the generation of realistic embryo images will be enhanced. (iii) Augmented training on Baseline model (AUG + Baseline): Incorporates data AUG strategies such as mirroring during training to enhance the diversity of the training data. This setup evaluates the impact of data AUG on Baseline model’s generative capabilities. (iv) Augmented and pretrained model (AUG + Pretrained-T): Combines pretrained weights from FFHQ with data AUG, facilitating the comprehensive assessment of both strategies’ combined impact on generating high-quality embryo images. (v) Augmented and pretrained model with translation and rotation equivariance (AUG + Pretrained-R): In addition to pretrained weights and data AUG, this design adds translation and rotation equivariance during training to explore how it interacts with pretrained weights and AUG to influence generative performance (Fig. 1b).

### Quantitative assessment

Fréchet inception distance (FID) (Heusel *et al.*, 2017) and kernel inception distance (KID) (Bińkowski *et al.*, 2018) are essential metrics employed in the field of generative modeling to quantitatively assess the quality and similarity between real and generated images. FID is based on the assumption that features extracted by a pretrained Inception-v3 network (Szegedy *et al.*, 2015), specifically at the pool3 layer, can be modeled as Gaussian distributions. It quantifies the resemblance between these feature distributions in real and generated data by computing the Fréchet distance between the corresponding multivariate Gaussian distributions:

$$\mathrm{FID}={∥{∥\mu }_{r}-\mu_{g}∥∥}^{2}+Tr\left(\Sigma_{r}+\Sigma_{g}-2{\left(\Sigma_{r}\Sigma_{g}\right)}^{1/2}\right)$$

where $N\left(\mu_{r},\Sigma_{r}\right)$ and $N\left(\mu_{g},\Sigma_{g}\right)$ are Gaussian distributions fitted to real and generated data, respectively. FID relies on the assumption that these features follow Gaussian distributions and measures their similarity. In contrast, KID offers a non-parametric approach to assessing image resemblance. It does not rely on any specific distribution assumptions, enhancing its versatility and robustness. KID complements FID by providing an alternative metric that captures image quality without assuming Gaussian distributions. Both FID and KID are valuable measurements for evaluating the performance of generative models and assessing how closely generated images resemble real data (Fig. 1c).

### Visual Turing test to assess quality of generated images

In addition to quantitative metrics, this study implemented a visual Turing test (Geman *et al.*, 2015) survey to evaluate the quality of the generated images. We sent out email invitations to perform the visual Turing Test, mainly targeted at clinicians/researchers working in the reproductive health field, those IVF fertility centers including Homerton Healthcare NHS Foundation Trust, Oslo University Hospital, Baltic Fertility Society, UZ Brussel, UZ Leuven, Amsterdam University Medical Center (UMC), UMC Utrecht, UMC Groningen, Radboud UMC, and Maastricht UMC. A total of 60 evaluators responded to the task of visually comparing both real and generated images of IVF blastocysts. These evaluators were categorized into three groups: Group I consisted of 25 experienced embryologists, with 19 individuals possessing over 5 years of expertise in assessing embryo quality; Group II comprised 15 IVF lab technicians, with 10 of them having more than 5 years of experience in working with IVF embryos; and Group III included 20 non-experts who had no experience on human embryo microscopic imaging. Each evaluator independently assessed a set of 100 blastocyst images, comprising 50 synthetic and 50 authentic images. True images were randomly selected from the training dataset, and the generated images were produced using random seeds. Evaluators operated without time constraints and were unaware of the distribution of synthetic and real images. They were presented with two choices to classify image fidelity: ‘Real’ or ‘Fake’. To facilitate the evaluation process, we created an online form to randomly present images to the evaluators (Supplementary Fig. S1). Evaluation metrics were computed based on the results of this visual Turing test, providing quantitative measures to the ability of participants to distinguish real from synthetic images, including accuracy, sensitivity, and specificity (Fig. 1d).

### Computational and statistical analysis

The computational resources used in this study included four Tesla V100 graphics processing units (GPUs) with 32 GB of memory each. Each model underwent 5000 iterations for training, which took 26 h to complete. For further refinement in image quality before undergoing the visual Turing test, the best model continued training to 25 000 iterations as default setting for StyleGAN3 training, requiring approximately 128 h (equivalent to 5 days and 8 h). For statistical analysis, a Kruskal–Wallis test was conducted for each metric (accuracy, sensitivity, and specificity) to assess significant differences among the three evaluator groups. Statistical analysis was performed using Python, with a significance level set at *P *<* *0.05.

## Results

### Artificial neural network architecture and (hyper)parameter configurations

We adapted a style-based GAN as baseline training model (Karras *et al.*, 2021), with superior performance which can be attributed to several key features: (i) the progressively growing GAN allows the generator to start with low-resolution images and progressively refine them, making it proficient at handling high-dimensional outputs, (ii) the use of an intermediate embedding space, combined with adaptive instance normalization and noise injection, which enhances image quality and diversity, and (iii) mixing regularization and noise injection further improve the robustness and diversity of generated images (Karras *et al.*, 2018). We distributed the training over four GPUs to accelerate the training process and handle the computational demands effectively. (Hyper)parameter settings are chosen with specific goals. Specifically, the use of ‘mirror’ for augmenting the dataset with random x-flips, which doubles the number of training images. Additionally, parameter ‘aug=ada’ allows adaptive discriminator augmentation (ADA), which stabilizes training in limited data regimes to against overfitting (Karras *et al.*, 2020). Based on the size of the training dataset and image resolution, we set the batch size as 32, with discriminator’s learning rate of 0.002, and gamma (R1 regularization weight) value of 2. The use of ‘freezed’ to freeze the first layers of discriminator was set to 13, according to experimental experience.

### Quantitative assessment via Fréchet inception distance and kernel inception distance

**Figure 3.**
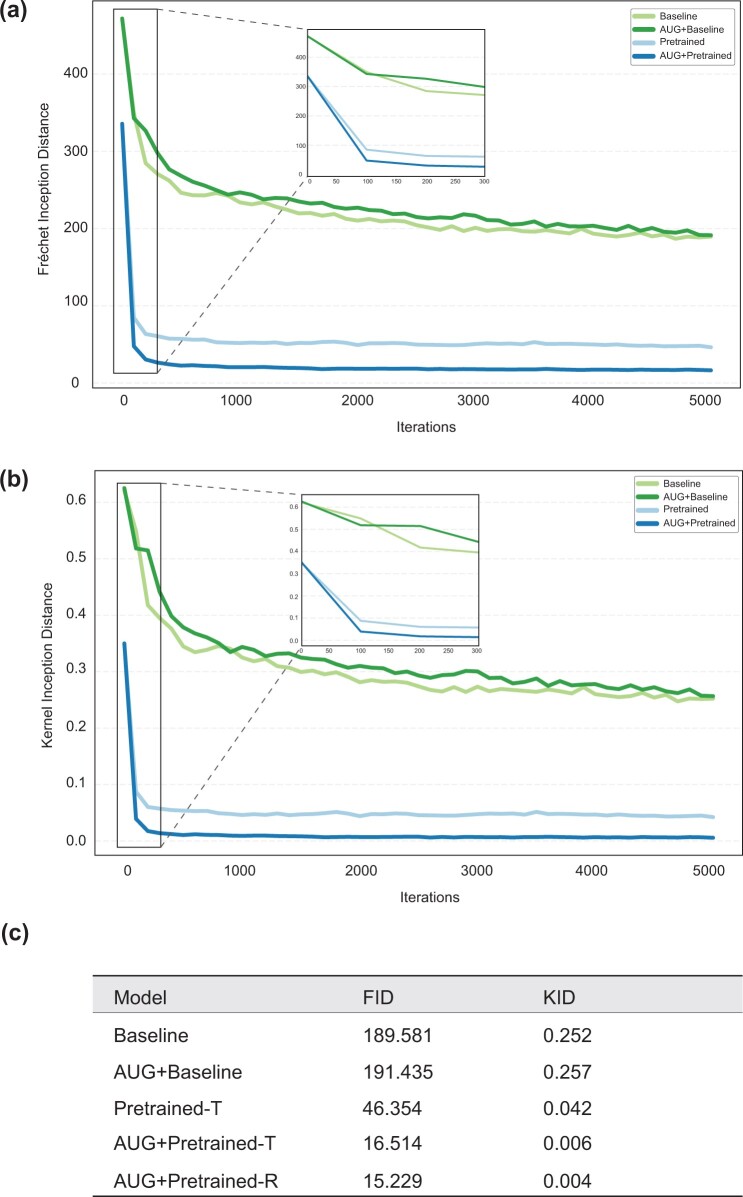
**Evaluation of generative models training results**. (**a**) FID values of four generative models for training 5000 iterations. (**b**) KID values of four generative models for training 5000 iterations. (**c**) Training results comparison in different models.

FID scores serve as a measure of the dissimilarity between generated and real images, with lower scores indicating better alignment. All models exhibited similar trends in their FID curves, demonstrating a gradual decline in FID values over successive training iterations (Fig. 3a). The Baseline and AUG + Baseline models started with a relatively elevated FID score of 472.0, indicating significant disparities between its initially generated and real images. However, as training progresses, FID value consistently diminished, reflecting a continuous enhancement in image quality. During training, both models experienced rapid FID reduction followed by stabilization. After 5000 iterations, FID of Baseline and AUG + Baseline model was 189.6, 191.4, respectively (Fig. 3c).

Conversely, both Pretrained and AUG + Pretrained initiated with a notably lower FID value of 336.0 compared to Baseline models. This suggests that models initialized with pretrained weights generate images that closely align with the distribution of real images from the outset. The FID value continued to decrease during training, indicating the substantial contribution of pretrained weights to generating high-quality images resembling real ones (Fig. 3a). The FID of Pretrained-T model after 5000 training iterations was 46.4. The FID of AUG + Pretrained-T and AUG + Pretrained-R was 16.5 and 15.2, respectively (Fig. 3c).

The KID scores serve as a compensatory metric for FID in assessing the resemblance between actual and generated images: lower KID scores indicate a closer resemblance. Across all models, consistent trends were observed in their KID curves, illustrating a gradual decrease in KID values as training progresses (Fig. 3b). In both Baseline and AUG + Baseline, the KID values underwent a steady reduction, indicating an improved ability of the model to generate samples close to real data. After 5000 iterations, KID of Baseline and AUG + Baseline is 0.252 and 0.257, respectively (Fig. 3c).

As training processes, the Pretrained and AUG + Pretrained models displayed a rapid decrease in KID values, signifying substantial advancements in generating more realistic samples (Fig. 3b). After 5000 iterations, the KID of Pretrained-T model was 0.042. The KID of AUG + Pretrained-T and AUG + Pretrained-R was 0.006 and 0.004, respectively (Fig. 3c).

### Qualitative assessment via visual Turing test

**Figure 4.**
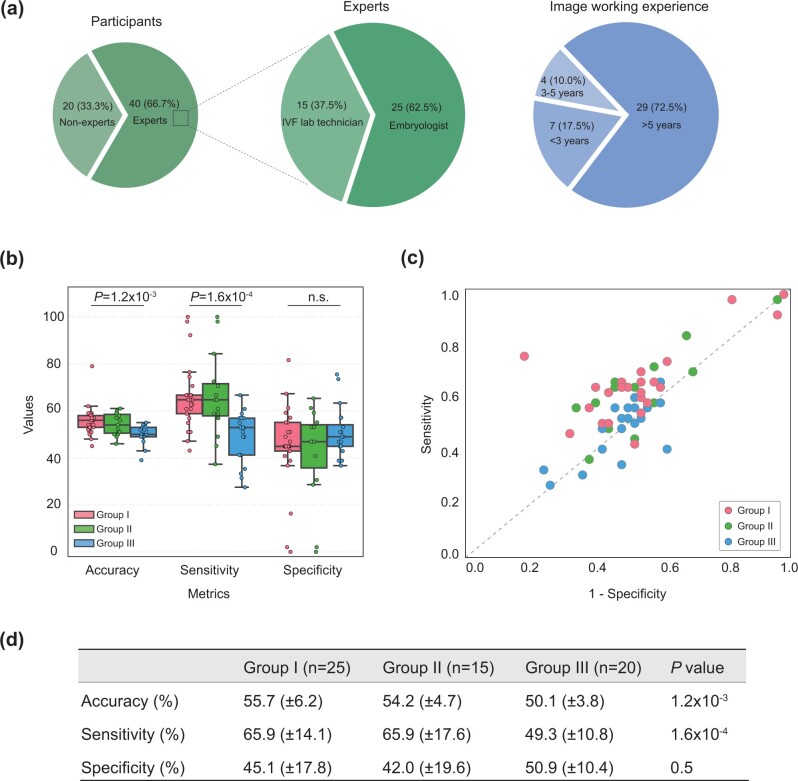
**Visual Turing test results from participants evaluation**. Group I: embryologists; Group II: IVF laboratory technicians; and Group III: non-experts. (**a**) Proportions of participants’ background and embryo microscopy/imaging working experience. (**b**) Distribution of evaluation metrics including accuracy, sensitivity, and specificity. (**c**) Visualization of positive likelihood ratio (Sensitivity/(1 − Specificity)), showing the likelihood of accurately distinguishing real and generated images within the three groups. (**d**) Evaluation metric results table. Kruskal–Wallis test was conducted for each metric to assess the statistical difference.

The AUG + Pretrained-R model achieved its optimal training results after 5000 iterations (Fig. 3c) and continued training up to 25 000 iterations to produce high-quality synthetic images for the visual Turing test. The image survey received a total of 60 responses by inviting participants to discriminate the true and synthetic blastocyst images. Inside the experts group (Group I and II), 72.5% had more than 5 years of experience working with IVF embryos, 10.0% with 3–5 years of experience and 17.5% of them having a maximum of 3 years of working with IVF embryos (Fig. 4a). For the visual Turing test results, Group I displayed accuracy of 55.7% (±6.2), sensitivity of 65.9% (±14.1), and specificity of 45.1% (±17.8). Group II showed 54.2% (±4.7) of accuracy, sensitivity of 65.9% (±17.6), and specificity of 42.0% (±19.6). Group III had the accuracy of 50.1% (±3.8), sensitivity of 49.3% (±10.8), and specificity was 50.9% (±10.4). Statistical analysis indicated significant differences in accuracy (Kruskal–Wallis test, *P *=* *1.2 × 10^−3^) and sensitivity (Kruskal–Wallis test, *P *=* *1.6 × 10^−4^) among the three groups, highlighting varying performance levels. No statistically significant difference was observed in specificity among three groups (Kruskal–Wallis test, *P *=* *0.5, Fig. 4b and d). We visualized the positive likelihood ratio (sensitivity/(1 − specificity)), which denotes the likelihood of accurately distinguishing real and generated images within the three groups (Fig. 4c). Group I, Group II, and Group III, depicted in red, green, and blue, respectively, demonstrated the positive likelihood ratio around the random guessing line (50%), underlining that the majority of the participants achieved a comparable accuracy with a random guess in discriminating between real and synthetic images.

### Interpretation of generated images

**Figure 5.**
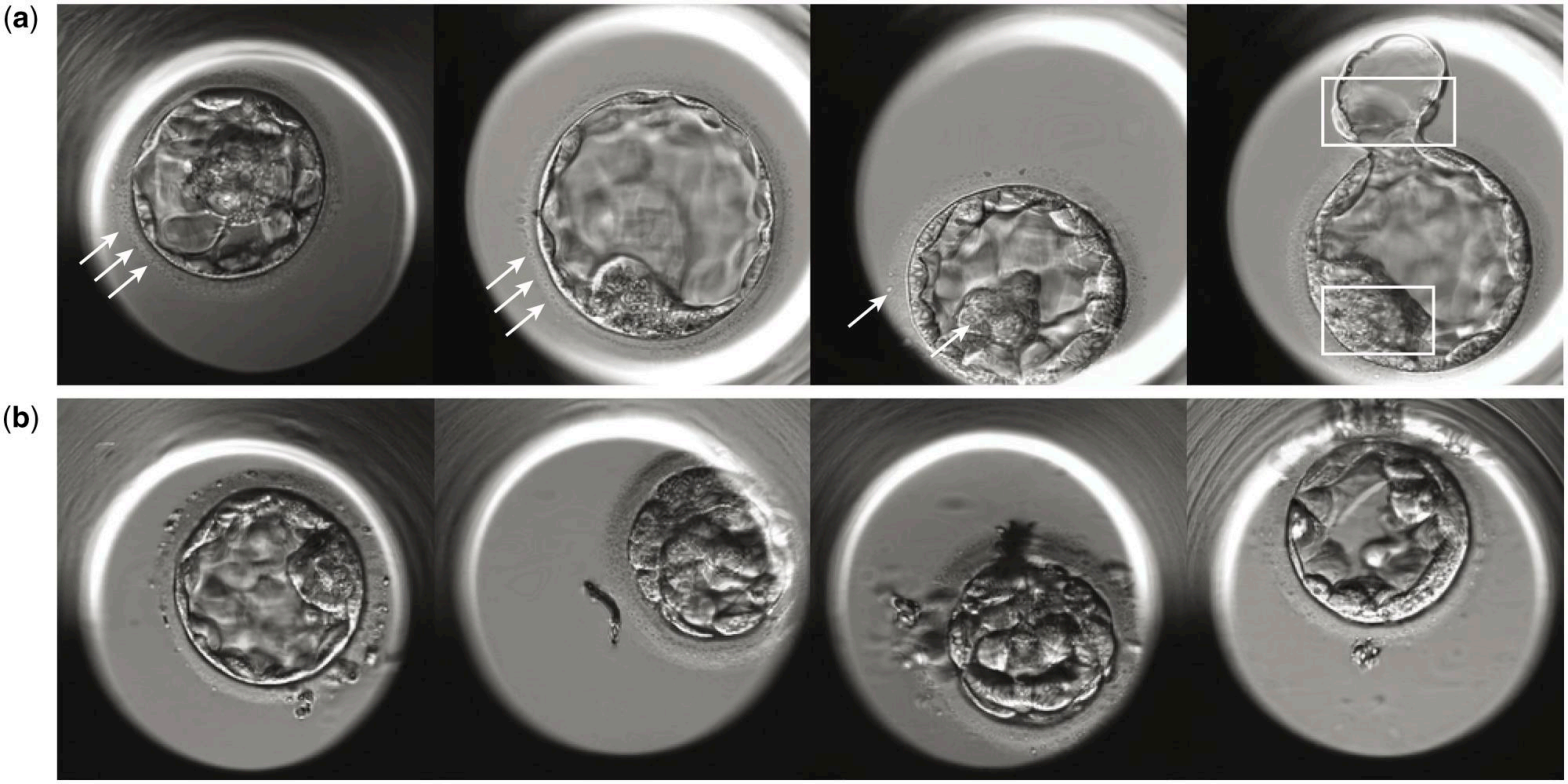
**Samples of generated images that were evaluated by participants**. (**a**) Generated images recognized as ‘Fake’ by 70% of embryologists (n = 18). White marks annotated as the obvious features that were considered artificial, including neatly arranged small bubbles in the *zona pellucida*, artificially looking white dots in the *zona pellucida* and inner cell mass, swirly structure in the inner cell mass, and distorted shape of the hatched cells (explained from left to right). (**b**) Generated images deceived 80% of embryologists (n = 20) that were not considered as fake images in the visual Turing test.

This section explains the interpretation of generated blastocyst images to gain insights into their fidelity and identify potential areas for improvement. We asked one experienced embryologist to report the key features on the generated images that were identified as fake by the majority of embryologists (70%, n = 18). Only 8% of generated images (4 out of 50) were detected as ‘fake’ by these experts following the visual Turing test due to features that were considered artificial (Fig. 5a). One of the most remarkable observations was the presence of neatly arranged small bubbles, white dots or artifacts within the *zona pellucida* that were perceived as artificial and inconsistent with true blastocyst images. Furthermore, hatched embryos presented unique characteristics that made them distinguishable from *in vitro* blastocysts. Specifically, the generated images of hatched blastocysts exhibited varying degrees of distortion in the hatched cells, setting them apart from the typical appearance of cells in real blastocyst images (Fig. 5a). Additionally, we presented generated images that managed to deceive a significant majority of experts (80%, n = 20), as they were not immediately recognized as fake images (Fig. 5b).

## Discussion

The development of AI-based methods has substantial potential to improve the understanding of embryogenesis and IVF clinical outcome. However, there have been limited publicly available datasets for developing AI methods to study embryo morphokinetics for embryo selection with the aim of improving IVF outcome. Therefore, we set out to explore the application of generative models to produce good-quality synthetic images for the purpose of developing robust AI models to select viable embryos. Here we demonstrate that adapting generative AI models, particularly a style-based GAN with pretrained weights and data AUG, substantially enriches embryo imaging datasets. This enrichment has a broad range of implications for advancing AI methods in embryo selection and improving clinical outcomes in IVF.

To date, AI-based models have been applied in embryo automatic grading (Chen *et al.*, 2019; Khosravi *et al.*, 2019), implantation prediction (Tran *et al.*, 2019; Bormann *et al.*, 2020; Geller *et al.*, 2021; Fordham *et al.*, 2022; Duval *et al.*, 2023), and ploidy prediction (Chavez-Badiola *et al.*, 2020; Huang *et al.*, 2021; Lee *et al.*, 2021; Diakiw *et al.*, 2022; Barnes *et al.*, 2023). While these models have shown promise in various aspects of IVF, ethical and privacy concerns, along with limited access to embryo imaging data, continue to challenge the efficacy of AI-based approaches to enhance embryo selection procedure and improve clinical outcomes.

In this study, we carried out both quantitative and qualitative assessments to test the performance of generative models. For quantitative evaluation, we employed FID and KID to objectively measure the dissimilarity between generated and true images. Both FID and KID consistently yielded lower values at 5000 iterations compared to previous medical research using generative techniques. For instance, generating lung CT images using StyleGAN achieved FID of 220.1 (Toda *et al.*, 2022), and FID results from liver, cardiac, and diabetic retinopathy datasets ranging from 23.7 to 29.1 (Skandarani *et al.*, 2021). In this study, the FID from the AUG + Pretrained-R model was 15.2 at 5000 iterations, exhibiting a decrease to 11.8 at 25 000 iterations. These findings provided valuable insights into the quality of the synthetic embryo images. In a visual Turing test involving 60 evaluators with three levels of embryo classification expertise including clinical embryologists, IVF lab technicians, and non-experts. Embryologists (Group I) and IVF lab technicians (Group II) achieved a higher accuracy than non-experts in distinguishing real from generated embryo images. However, the accuracy rate for embryologists (Group I) is only 55.7%, compared to 61.3% from assessing synthetic gastroscopy images (Shin *et al.*, 2023), and 67.4%, 69.9% from evaluating two sets of generated chest radiographs (Jang *et al.*, 2023). Additionally, in this study, individuals without specialized expertise in embryo imaging performed at a level approximately equivalent to random guessing (50%). These results underscore the ability of the generative model, as it poses a substantial challenge to human visual discrimination. Additionally, we provided a resource of synthetically generated images (n = 5000) that facilitate future AI-based embryo selection model developments.

To the best of our knowledge, this is the first study generating blastocyst images tackling embryo imaging scarcity, and the first study involving a great number of human participants (n = 60) both experts and non-experts for evaluating generated images. The synthesis of human embryo images using generative models presents great promise in developing AI methods in reproductive medicine. Primarily, it could provide extensive data where synthetic embryo images exhibit diverse developmental characteristics. This would substantially enhance the training data, empowering AI-based methods to excel in the assessment of embryo quality and developmental potential. Particularly, generative models may provide sufficient data in rare (abnormal) events during embryogenesis, such as tripolar cell division. Second, the incorporation of generated images paves the way for the development and validation of innovative embryo scoring systems. These systems could integrate dynamic morphological features, offering the potential for more comprehensive and precise assessments. Ultimately, this can lead to enhanced embryo selection procedures and might improve success rates in IVF treatments.

This study has some limitations. The training data only focused on blastocyst-stage embryo images because blastocysts represent the stage at which embryologists often assess both the morphological quality of embryos and perform cell biopsies for preimplantation genetic testing. Furthermore, it has been reported that the 100–120 hpi duration during embryo development holds the highest predictive value when employing AI-based methods (Erlich *et al.*, 2022). To address the scarcity of data and contribute to advancements in embryo selection procedures, we utilized generative models focused on blastocyst images, generating a diverse array of such images to offer insights for future AI studies, i.e. morphokinetics annotation, blastocyst segmentation, and automatic grading tasks. However, it is also necessary for future research to broaden its scope by incorporating a more extensive and diverse dataset, with the aim of facilitating the development of comprehensive AI algorithms. For instance, the generation of the whole time-lapse video could provide more dynamic captures and facilitate the understanding of embryogenesis. This enriched data could help develop robust data-driven approaches to advance automation in IVF. Another limitation is the resolution of 256 × 256 pixels that we used to mitigate computational costs and given the resource-intensive nature of training StyleGAN. Subsequent research will be conducted to improve the image resolution to provide supreme-quality of generated images. Future work is needed to provide evidence on elucidating the benefits derived from data enrichment through generative models, such that data augments from generated images could enhance the performance and robustness of AI algorithms for embryo selection. In addition, integrative clinical, e.g. pregnancy and live-birth outcomes, and imaging AI-models are required to illuminate the clinical impact of GAN in embryo selection procedures.

In conclusion, in this pilot study, we demonstrated that the generative models have the capacity to generate high-fidelity human embryo images, indicating the potential of generative AI in revolutionizing embryo selection and advancing IVF procedures.

## Supplementary Material

deae064_Supplementary_Data

## Data Availability

The image training dataset, generated blastocyst image dataset, pretrained weights, and visual Turing test survey in this study are available online at https://github.com/CellularGenomicMedicine/StyleEmbryo. Training scripts, user instructions, and results, including visualization notebooks are available in the same repository.
