# Supplementary material for: Generative artificial intelligence to produce high-fidelity blastocyst-stage embryo images
Source: Hum Reprod. 2024 Apr 10;39(6):1197–207. doi: 10.1093/humrep/deae064 (PMC11145014; doi:10.1093/humrep/deae064)
Supplement: deae064_Supplementary_Data [file deae064_supplementary_data.pdf]

Human Reproduction, 2024, 1–1
https://doi.org/10.1093/humrep/deae064
Supplementary Data

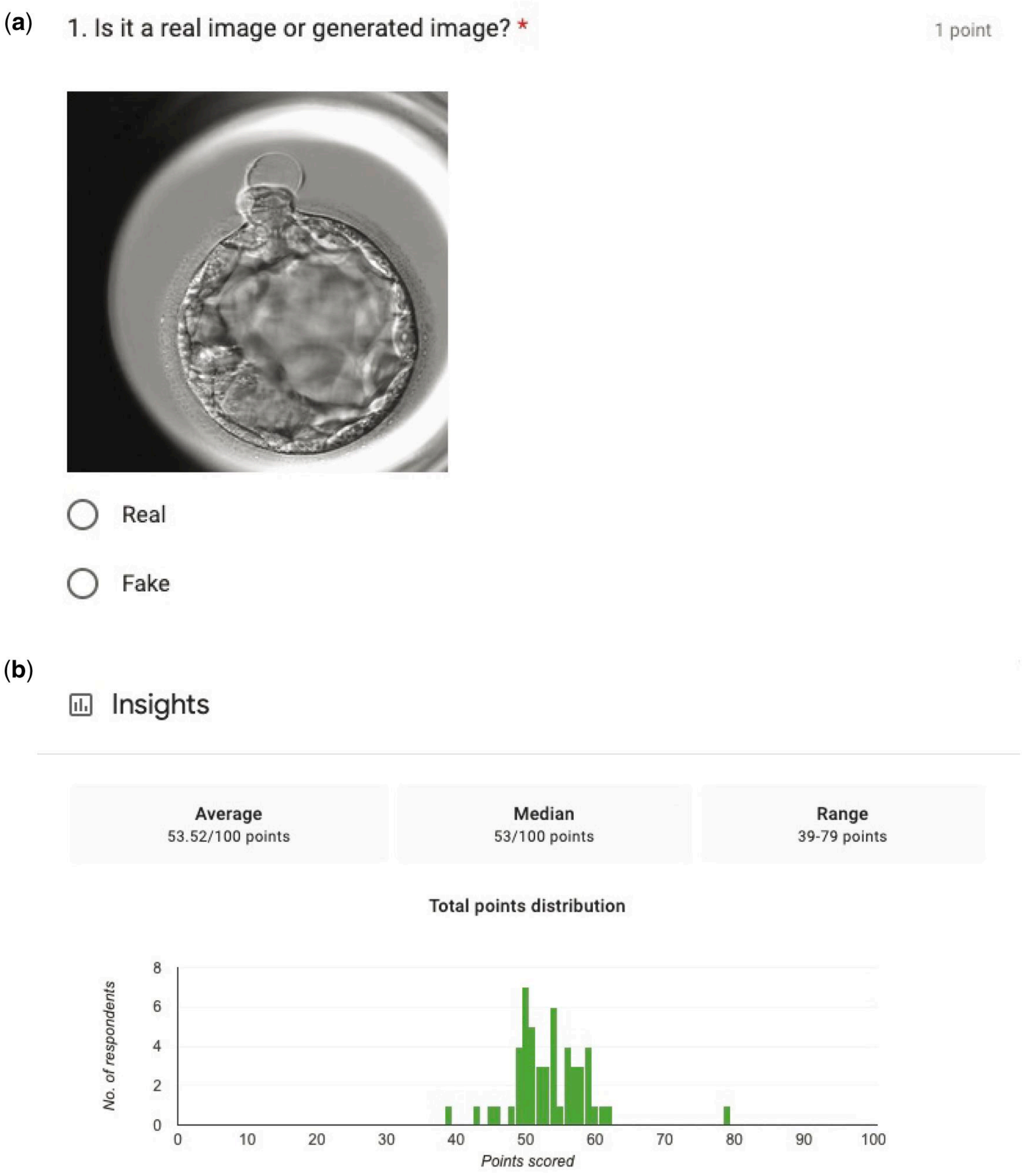

Supplementary Figure S1. Screenshot of image visual Turing test survey. (**a**) Interface for participants to distinguish the real images from the generated images, their responses were collected after submission. (**b**) Interface for operators to analyze the responses received from this survey. More information could be seen from the Google Form link at https://forms.gle/NwX9ZbYhxXZjvhEz7.

© The Author(s) 2024. Published by Oxford University Press on behalf of European Society of Human Reproduction and Embryology.
Human Reproduction, 2024, 00, 1–12
https://doi.org/10.1093/humrep/deae064
Original Article
